# Optical Thin Films Fabrication Techniques—Towards a Low-Cost Solution for the Integrated Photonic Platform: A Review of the Current Status

**DOI:** 10.3390/ma15134591

**Published:** 2022-06-29

**Authors:** Muhammad A. Butt, Cuma Tyszkiewicz, Paweł Karasiński, Magdalena Zięba, Andrzej Kaźmierczak, Maria Zdończyk, Łukasz Duda, Malgorzata Guzik, Jacek Olszewski, Tadeusz Martynkien, Alicja Bachmatiuk, Ryszard Piramidowicz

**Affiliations:** 1 Institute of Microelectronics and Optoelectronics, Warsaw University of Technology, Koszykowa 75, 00-662 Warszawa, Poland; andrzej.kazmierczak@pw.edu.pl (A.K.); ryszard.piramidowicz@pw.edu.pl (R.P.); 2 Department of Optoelectronics, Silesian University of Technology, ul. B. Krzywoustego 2, 44-110 Gliwice, Poland; cuma.tyszkiewicz@polsl.pl (C.T.); pawel.karasinski@polsl.pl (P.K.); magdalena.zieba@polsl.pl (M.Z.); 3 Lukasiewicz Research Network-PORT Polish Center for Technology Development, Stablowicka 147, 54-066 Wroclaw, Poland; maria.zdonczyk@port.lukasiewicz.gov.pl (M.Z.); lukasz.duda@port.lukasiewicz.gov.pl (Ł.D.); malgorzata.guzik@port.lukasiewicz.gov.pl (M.G.); alicja.bachmatiuk@port.lukasiewicz.gov.pl (A.B.); 4 Faculty of Chemistry, University of Wrocław, ul. F. Joliot-Curie 14, 50-383 Wrocław, Poland; 5 Department of Optics and Photonics, Faculty of Fundamental Problems of Technology, Wroclaw University of Science and Technology, Wybrzeze Wyspianskiego 27, 50-370 Wroclaw, Poland; jacek.olszewski@pwr.edu.pl (J.O.); tadeusz.martynkien@pwr.edu.pl (T.M.)

**Keywords:** sol-gel dip-coating method, chemical vapor deposition, silica-titania waveguide platform, ion exchange, ion implantation, electron beam evaporation

## Abstract

In the past few decades, several methods concerning optical thin films have been established to facilitate the development of integrated optics. This paper provides a brief depiction of different techniques for implementing optical waveguide thin films that involve chemical, physical, and refractive index modification methods. Recent advances in these fabrication methods are also been presented. Most of the methods developed for the realization of the thin-films are quite efficient, but they are expensive and require sophisticated equipment. The major interest of the scientists is to develop simple and cost-effective methods for mass production of optical thin films resulting in the effective commercialization of the waveguide technology. Our research group is focused on developing a silica-titania optical waveguide platform via the sol-gel dip-coating method and implementing active and passive optical elements via the wet etching method. We are also exploring the possibility of using nanoimprint lithography (NIL) for patterning these films so that the fabrication process is efficient and economical. The recent developments of this platform are discussed. We believe that silica-titania waveguide technology developed via the sol-gel dip-coating method is highly attractive and economical, such that it can be commercialized for applications such as sensing and optical interconnects.

## 1. Introduction

The field of integrated optics is evolving rapidly, and the ultimate objective is to establish small-scale optical circuits comparable to the silicon chips that have transformed the electronics industry. The benefit of the optical approach is that data can be managed at much higher speeds [1]. Fiber-optic communication networks [2], commercial telecommunications [3], military communications [4], and computer-to-computer data communication links [5] are some of the general spheres where integrated optics provide performance-boosting advantages [6]. Additional valuable applications are projected in the fields of optical sensing [7,8], optical signal processing [9], and perhaps optical computing [10].

There are several optical platforms available for the realization of optical interconnects, such as semiconductor waveguides (hereafter represented as WGs) [11,12,13], dielectric WGs [14,15,16], crystalline WGs [17,18,19], polymer WGs [20], glass optical WGs [21,22], plasmonic WGs [23,24,25] and hybrid plasmonic WGs [26,27,28], among others. One must choose the right platform for the particular purpose based on the ease of fabrication, practical applicability, optical losses, and footprint. In the last few decades, several eye-catching techniques have been developed to implement high-quality optical thin films for light-guiding applications [29]. Thin films are the foundation for innovative technologies in various areas, including optical devices, environmental applications, telecommunications devices, and energy storage devices [30]. The morphology and reliability of thin films are critical issues in all applications. Deposition techniques have a major influence on thin-film morphology. Physical and chemical deposition methods can be used to deposit high-quality thin films. A thin film is a thin layer of material with a thickness ranging from a few nm to a few μm. Thin films, like all materials, are classified as amorphous or polycrystalline based on the preparation conditions and the quality of the target material.

Glasses are ideal platforms for implementing active, passive, and nonlinear optical components due to their exceptional chemical and physical characteristics, specifically their optical attributes, such as wide wavelength operational range and an elevated threshold for optical damage [31]. Specifically, silica glasses are highly desirable as they complement the fiber optics entirely and introduce minimal coupling losses and high thermal and mechanical stability. Furthermore, these glasses have a very low expansion coefficient on silicon, allowing for the deposition of thick buffer layers, which are a key element on silicon for WG purposes [21]. The SiO_2_/Si technology, which allows silica glass-based WGs to be produced using various methods, is particularly useful. Flame hydrolysis deposition (FHD) [32,33] and chemical vapor deposition (CVD) [34] offer exceptional thin film quality and have been widely studied. The sol-gel process offers countless benefits such as avoiding complicated apparatus, and high-cost fabrication processes are a substitute solution to these manufacturing methods [35].

**Figure 1:**
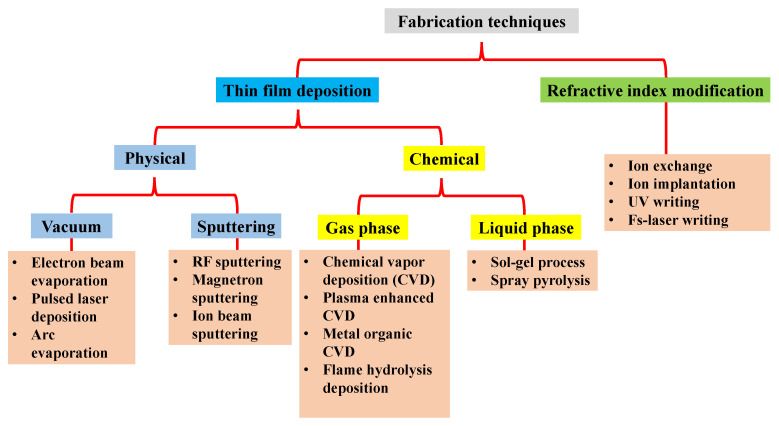
A layout of most widely used techniques to fabricate glass optical WGs. Inspired by [21].

Glass WGs display highly attractive properties due to the straightforward technology, the low propagation losses, and the flexible index matching to glass fibers. It is highly desirable to have low-loss glasses, reliable and enabling low-cost WG fabrication procedures. An overall requirement is that manufacturing technologies are proficient in high yield, and have guaranteed duplicability within the quantified tolerances, and fundamentally low operating costs. Subject to the glass integrated optics, it is conceivable to classify the most common WG manufacturing techniques according to Figure 1. The fundamental requirement for light confining is that the guiding layer should have a higher refractive index than the substrate and cladding [16,17]. As a result, two main approaches can be practiced implementing the guiding layer, i.e., thin-film deposition and local adjustment of the bulk material. Various processes, for instance, RF-sputtering and magnetron sputtering, CVD, plasma-enhanced CVD, FHD, spray pyrolysis deposition, pulsed laser deposition, and the sol-gel coating are included in the thin layer deposition category. Whereas local modification of the bulk material can be performed with ion exchange, ion implantation, or UV-irradiation, the fs-laser writing is also appropriate for the direct inscription of a channel WG in the substrate.

This review paper is structured in the following manner: In Section 2, optical WG thin-film deposition methods are discussed, including physical, chemical, and local refractive index modification. Then, several deposition techniques and the recent advancements in each method are presented. Our goal is not to give the final verdict on the best deposition technique. Instead, the main purpose is to describe the advantages/disadvantages associated with each deposition technique. We leave the choice to the readers to decide which method is suitable for their needs. Indium phosphide (InP), silicon-on-insulator (SOI), and silicon nitride (SiN) are three popular PIC technologies that have reached industrial competence. Fabrication of PICs with these technologies requires sophisticated and expensive equipment, since it often necessitates gaseous phase WG film deposition (using, for example, the LPCVD process), stringent lithography (using E-beam or deep VU techniques), and plasma etching (RIE or ICP). This makes it hard for smaller research institutes, colleges, or SMEs to apply these technologies directly. Section 3 presents the highlights of the silica-titania (SiO_2_-TiO_2_) platform developed via the sol-gel dip-coating method. This platform is highly attractive due to its excellent optical, physical and chemical properties. Moreover, the production cost of high-quality thin-films is quite low. Our research group has been working on developing this platform for several years and the possibility of patterning these thin films via nanoimprint lithography (NIL) is being explored. The current achievements in this topic are documented along with the previous literature review. Finally, this platform’s future goals and challenges are presented in Section 4, followed by a brief conclusion in Section 5.

## 2. Optical Waveguide Layers Fabrication Methods

Waveguiding depends on the adequate refractive index contrast between the guiding layer and the substrate/upper cladding [36]. Several techniques have been developed to produce optical thin films with a refractive index higher than the substrate. As a result, the light can be guided with the help of total internal reflection. According to the requirement, the WG layer is etched to obtain different WG types such as rib [37], ridge [38], and slot WG [39]. To avoid the etching process after the layer deposition, another method is developed, which provides the local modification of the refractive index of the substrate. This section discusses the most widely used WG fabrication methods with recent advances. Each method has distinct benefits and drawbacks, and no specific method can be believed to be better. The choice of a particular WG fabrication technique depends on the desired application and the available resources.

### 2.1. Thin-Film Deposition Techniques

Several physical and chemical deposition methods have been used to manufacture nanostructured thin films over the last two decades [29]. Both approaches have some benefits over traditional techniques and exhibit promising potential for the deposition process. Thin film technology requires the deposition of new transparent materials on conducting and non-conducting substrates at low temperatures. A thin film layer is a material with a thickness ranging from a fraction of a nanometer to a micrometer [40]. Physical vapor deposition (PVD), chemical vapor deposition (CVD), and refractive index modification techniques that allow for the deposition of such layers are considered in this paper.

#### 2.1.1. Physical Vapor Deposition Techniques (PVD)

Thermal evaporation method [41], electron beam evaporation [42], pulsed laser evaporation [43], molecular beam epitaxy [44], ion plating [45] and activated reactive evaporation [46] are all examples of PVD methods. This deposition process aims to move atoms from a source to a substrate, where film-forming and growth can happen independently. However, there are some disadvantages, such as the need for a tightly controlled vacuum environment and expensive instrumentation. PVD is a vaporization coating technique that requires an atomic-level material transfer. It is a vacuum-based process in which vaporized material from a source is transferred through a vacuum or low-pressure gas atmosphere to a substrate, where it condenses.

*A*. *Vacuum*

Electron beam (E-beam) evaporation is a type of PVD in which a charged tungsten filament emits an E-beam that bombards a target anode in a vacuum and allows atoms from the target to transition to the gaseous state. The atoms then solidify, leaving a thin anode material layer on everything in the vacuum chamber. E-beam deposition achieves a high deposition rate (0.1 μm/min to 100 μm/min) at low substrate temperatures while optimizing material utilization [29]. In addition, the deposition process allows for flexible tailoring of film structure and morphology, as well as the other desired material properties like dense coating, high thermal performance, low contamination, high durability, and high throughput [42].

The deposition chamber is pumped down to ~10^−5^ Torr pressure. Ingots or a compressed solid are utilized as the materials to be evaporated. The E-beam can be produced by thermionic emission, field electron emission, or the anodic arc method using electron guns. The E-beam is fast-tracked to high kinetic energy and is directed towards the target material. The electrons’ kinetic energy is converted to thermal energy, which increases the surface temperature of the materials, causing evaporation and deposition on the substrate. The temperature of the electrons is usually approximately 3000 °C, and they are accelerated towards the target material by a 100 kV DC voltage source. The deposition rate is determined by the starting material and the strength of the E-beam. The vapor pressure must be around 10 mTorr for adequate deposition rates. Several materials are practically hard to evaporate by thermal evaporation [47]. E-beam evaporation must be used instead to evaporate refractory metals.

**Figure 2:**
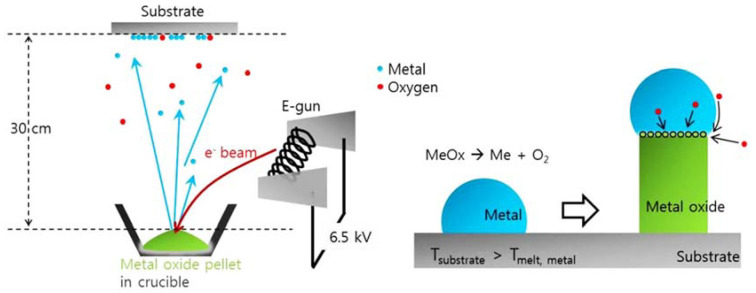
Diagrams of the E-beam evaporation model for the growth of metal oxide nanowire and growth process by vapor-liquid-solid [48].

In [48], the growth process of metal oxide nanostructures produced by E-beam evaporation is demonstrated. The condensed E-beam can simply decompose metal oxide sources that have a high melting point, thus forming self-catalytic metal nanodots for the vapor-liquid-solid (VLS) method. Figure 2 shows the E-beam evaporation model for the growth of metal oxide nanowire. One benefit of E-Beam Evaporation is the possibility to rotate various source materials into the electron’s path, allowing many thin films to be deposited successively without disrupting the vacuum. A wide range of materials are deposited using E-beam evaporation, which is extensively utilized for optical thin film applications ranging from laser optics and solar panels to eyeglasses and architectural glass. It offers the necessary optical, electrical, and mechanical properties. When compared to other PVD processes, E-beam evaporation has a high material use efficacy, lowering the manufacturing cost. Various high-quality thin films such as stainless steel [42], copper [49], silicon [50], silicon nitride [51], and many more [52,53,54,55] have been deposited via the E-beam deposition method.

Another physical deposition method for the thin-film-coating system is pulsed-laser deposition (PLD), in which the laser beam is used to ablate the target material for depositing thin films in a vacuum chamber [56]. Different types of laser sources are used to ablate the target, such as Nd-YAG laser, KrF (248 nm), and XeCl (308 nm). When a laser beam hits a target material, it creates a plume that can be deposited on several substrates. The plume can be composed of ionized species as well as neutral and ground-state atoms. To acquire metal oxide thin films, oxygen is used in the process. The quality of thin film deposited by the PLD method is determined by various factors, including the wavelength of the laser, energy, atmospheric gas pressure, pulse size, and the target-to-substrate distance [57]. As illustrated recently, PLD has been used to deposit an extremely diverse variety of materials. The most important use of PLD in the past has been demonstrated in high-temperature superconducting thin films. The experiment revealing that PLD could be used to deposit YBa_2_Cu_3_O_7−x_ (YBCO) films with zero resistivity at nearly 85 K triggered intensive research on the high-temperature superconductivity over the last decade, as well as on PLD in general [58]. For detailed knowledge about the PLD method, please consult [43].

The mechanism of cathodic arc deposition (CAD) has been the subject of extensive study in recent years [59]. This method has unique properties that can be effectively applied in both basic and applied physics, especially in implementing functional coatings. Several works explain the benefits, shortcomings, and current state of understanding about CAD today [60,61,62]. A discharge of electricity between two electrodes is utilized to create a coat in this method. The evaporation mechanism starts with striking a high current, low voltage arc on the surface of a cathode, which produces a thin, highly energetic emitting zone known as a cathode spot. The temperature at the cathode spot is exceptionally high (around 15,000 °C), resulting in a jet of vaporized cathode material moving at a high velocity (~10 km/s). The cathode spot is only active for a brief period before self-extinguishing and re-igniting in a different region near the previous spot. This behavior causes the visible motion of the arc. When a reactive gas is released during the evaporation process, it may cause dissociation, ionization, and excitation when it interacts with the ion flux, resulting in the formation of a compound film. Industry compatibility, good film adhesion, excellent stoichiometric control, low temperature, multilayer compact coating, uniform film, and low voltage are all benefits of this approach. However, it has the drawback of being unable to deposit complex structures. Several functional layers have been coated via the CAD method for integrated photonics applications [63,64,65,66].

*B*. *Sputtering techniques*

Sputtering is a physical vapor deposition procedure with a high film deposition rate and low-temperature structures, making it a good technique [67,68]. It is fast and inexpensive to create thin films of alloys, metals, nitrides, carbides, and oxides [69,70,71]. The magnetron sputtering technique, which uses a magnetic field to support the process of depositing thin films onto a substrate, is the most common procedure for this technique [72]. Via the momentum transfer from the argon (Ar) ions, the particles (atoms and ions) are discharged. Electrons are confined to the magnetic field lines in magnetron sputtering. The target is bombarded with a gaseous plasma that keeps electrons and is then guided to grind down the material and expel them in the shape of neutral particles and a small portion of ions. An inactive gas such as Ar, or even an active gas, such as nitrogen, is widely used as sputter gas. The expelled particles would then settle on the substrate and form a thin layer of the target material.

Magnetron sputtering has many benefits over other methods, including uniformity, smoothness, and strong adhesion deposition over a very substantial region. The ability to select substrate and target materials with high melting points and a high deposition rate enables simple manipulation of deposited layer thickness [73]. However, the reactive sputtering method has a variety of drawbacks, including target poisoning, low deposition rates, and arcing that produces imperfections in thin films [74]. In the sputtering techniques, however, several key factors are employed to adjust the thickness of the manufactured films. The integrated pulse energy, time of deposition, pressure in the chamber, plasma gas, target-to-substrate angle, and substrate temperature are all crucial elements in diminishing dopant redistribution and defect creation during high-temperature processing. There are several types of high-quality thin films, such as boron carbon nitride [68], aluminum oxide [75], gallium oxide [76], and others [77], deposited via the magnetron sputtering technique [67]. For readers interested in the history of the thin-film sputter deposition process, we recommend consulting the reference [78].

Ion beam sputtering or ion beam deposition (IBD) employs an ion source to create a narrowly focused ion beam (FIB) aimed at the target material to be sputtered. Both the cathode and anode are concentrically aligned in the ion source. An electrostatic field is generated within the ion source when a high voltage field of 2–10 kV is applied, confining electrons in the center of the source [79]. As Ar gas is pumped into an ion gun, the high electric field causes the gas to ionize, resulting in plasma within the source region. Then the ions are fast-tracked from the anode area to the cathode exit aperture, resulting in a parallel ion beam. The resultant ion beam collides with a target material and sputters it towards the substrate due to momentum transfer between the ion and the target. The deposition procedure can be assisted with a second ion gun by bombarding the growing film, which is commonly known as ion beam assisted deposition (IBAD), which boosts adhesion, density, stoichiometry control, and low optical absorption at short wavelengths [80,81]. IBD, like other PVD processes, offers benefits such as deposition rate, homogeneity, composition, thickness control, adhesion, and material characteristics. IBD, on the other hand, has several advantages, including the ability to deposit a wide variety of materials regardless of target thickness or properties, precision deposition stops, clean and low-pressure processing (0.5 mTorr), and reactive deposition that is not vulnerable to high energy arcs due to cathode target poisoning.

There is no plasma between the substrate and the target in the IBD process, as there is in magnetron sputtering. This means that the IBD process may be utilized to deposit on sensitive materials, lowering the risk of gas molecules in the final product. IBD is highly suited for applications such as precision optics because of its controllability. The readers interested in the detailed explanation of the IBD technique are highly encouraged to read [82].

#### 2.1.2. Chemical Deposition Techniques

In chemical deposition techniques, materials to be deposited are permitted to respond to different chemicals, thereby permitting reactions to occur in a way that high-quality thin films are successfully deposited. Chemical deposition techniques involve gas-phase and liquid-phase deposition methods as discussed in the following section.

*A*. *Gas-phase*

Chemical vapor deposition (CVD) and flame hydrolysis deposition (FHD) techniques fall in the category of the gas-phase deposition method, which is discussed below:

*A.1. CVD techniques*

CVD is a deposition process that produces high-performance solid materials, usually under a vacuum. In this technique, chemical reactions between organometallic or halide compounds and other gases form the nonvolatile solid thin films that are deposited on substrates. The key difference between this method and PVD is that the material deposition on the substrate is multidirectional, while PVD is a line-of-site impingement. CVD is widely used in microfabrication processes to deposit materials in numerous forms such as epitaxial, amorphous, monocrystalline, and polycrystalline. Unlike PVD, a definite chemical reaction between a mixture of gases and the bulk surface of the material occurs in CVD, allowing the chemical decomposition of some of the components of the gas, establishing a solid coating on the surface of the substrate. In addition, this enables the structures and properties of the resulting products to be tuned [83], and several cutting-edge CVD systems and their alternates, for instance, plasma-enhanced CVD (PECVD) [84] and metal-organic CVD (MOCVD) [85], have been industrialized. Typically, high-vacuum working environments are not needed for CVD, enabling a predominant technology for electronics, optoelectronics, and biomedical applications. There are several reports on high-quality waveguide films deposited via CVD or its alternates [86,87,88]. A recently published brief review on CVD [88] should be studied for more details.

PECVD is a technique for depositing thin films of different materials on substrates at a lower temperature than traditional CVD. It is a hybrid coating technique in which energetic electrons trigger CVD processes (100–300 eV) inside the plasma rather than thermal energy, as in traditional CVD techniques. It is a vacuum-based deposition process that works at pressures as low as 0.1 Torr, permitting film deposition at substrate temperatures as low as 350 °C. There are several publications on the PECVD technique for the deposition of fine optical layers [89,90,91]. Since PECVD needs comparatively low substrate temperatures and offers high deposition rates, the films can be deposited onto large-area substrates that cannot survive the high temperatures needed by conventional CVD techniques. Thick coating >10 μm can be deposited with a different thermal expansion coefficient without stresses developing during the cooling [92].

MOCVD is a commonly used technique for a wide variety of materials, including electronic, optoelectronic, piezoelectric, ferroelectric, and multiferroic. Precursors made up of complex metal-organic ligands of metal ions are treated as a deposition source in this method. As opposed to PVD methods, one of the major benefits of MOCVD is that the precursors are held outside the deposition chamber and can thus be refilled continuously during long deposition runs. Several precursor mixtures with varying amounts of dopants can also be made quickly and effectively. Furthermore, superconductor film growth has been achieved at high deposition rates of more than 0.5 μm/min. Additionally, numerous mixtures of various precursors can be made with varying levels of dopants very quickly and effectively. Likewise, high deposition rates of more than 0.5 μm/min have been achieved in superconductor film growth [93]. MOCVD of other materials uses rates of about 1 μm/h, so such high rates are rare. Since MOCVD precursors can be deposited over a wide area at high deposition rates, high throughput is possible, which is crucial for large-scale manufacturing.

*A.2. Flame hydrolysis deposition (FHD)*

An FHD process is widely used to depose the WG films since it can provide an elevated deposition rate resulting in minimal optical losses and low-stress thin films [33]. In this deposition process, vapor precursors are added in a flame and experience chemical reactions to create soot particles which are afterward thermophoretically collected to form a porous layer on a substrate. To obtain a WG core, the porous soot deposited on the substrate must form a dense glass. To prevent the mixing of the layers, sintering temperatures are preferred such that the layers adjacent to the substrate have higher viscosities for the given temperatures. These temperatures are, sadly, often sufficient to allow the dopants to volatilize on the deposit surfaces. Consequently, in the densified glass layer, dopant concentration gradients and compositional inhomogeneities are formed, producing substandard optical properties. Such issues can be reduced or eliminated if the layers can be pre-sintered during deposition. In [32], pre-sintered WG glass films are created, allowing straight WG losses to diminish from ~0.3 dB/cm to ~0.05 dB/cm. To minimize layer mixing, sintering temperatures are adjusted so that the layers closest to the substrate have greater viscosities for a specific temperature. The FHD method offers the benefits of depositing glass films at high rates with minimal losses and producing films with low strain. However, the sintering temperatures for these layers are still high enough to induce the dopants to volatilize at the deposit’s surfaces, which is a concern. As a result, in the densified glass layer, dopant concentration gradients and compositional inhomogeneities form, resulting in poor optical characteristics. These issues might be lessened or eliminated if the layers could be placed pre-sintered during laydown. Several studies on the formation of low-loss planar WGs utilizing the FHD process have already been reported [33,94,95,96].

*B*. *Liquid-phase deposition*

Liquid-phase deposition (LPD) is a distinctive soft solution process achieved by uncomplicated techniques. The two most widely used methods are discussed in this section: spray pyrolysis and the sol-gel method.

*B.1. Spray pyrolysis method*

Spray pyrolysis is a method for preparing thin and thick films, ceramic coatings, and powders currently being researched [97]. Unlike many other film deposition techniques, it represents a very simple and relatively cost-effective processing method, especially concerning equipment costs. In this method, a thin film is deposited by spraying a solution on a heated surface where the constituents react to form a chemical compound [98]. Chemical reactants are chosen so that products other than the compounds required are volatile at the deposition temperature. It provides an incredibly simple technique to prepare films of any composition. Researchers have used this method to develop high-quality thin films which can be employed in different optical components [99,100,101,102]. Spray pyrolysis, on the other hand, has the fatal flaw of forming porous and/or hollow-structured particles [103,104]. A lot of research has gone into solving this problem [105,106]. The impact of salt solubility and physical properties on the morphology of zirconia particles made from five different salts was examined, finding that those made from precursor solutions with lower initial relative solution saturations established solid ZrO_2_ particles, while others formed hollow ones [107]. The inclusion of H_2_O_2_ in the ZrO_2_ particle preparation process aided solid particle production by slowing the breakdown reaction and preventing the development of the surface crust [108]. By introducing a stable colloidal solution into spray pyrolysis, multicomponent oxide particles with a spherical and dense shape were formed, in which the colloidal seed triggered the volume precipitation of droplets by serving as nucleation seeds [109].

**Figure 3:**
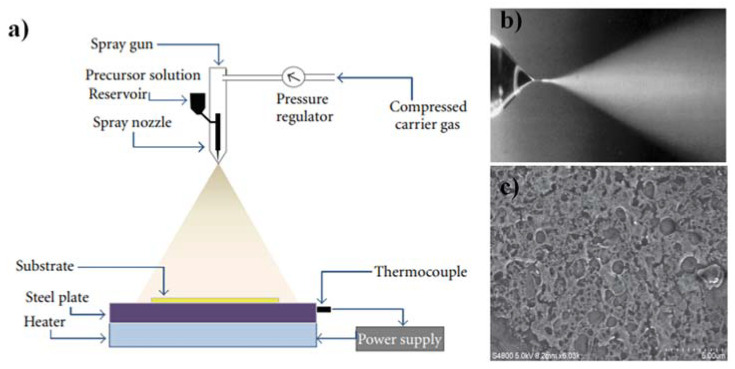
(**a**) The schematic of the spray pyrolysis setup [99], (**b**) Cone-jet spraying of methanol containing a small amount of HCL. Reprinted with permission from [110], (**c**) FESEM micrograph of SnO_2_ thin film [99].

Spray pyrolysis does not necessitate the use of costly substrates or chemicals. Instead, the process has been used for depositing thick films, porous films, and powder products. Using this flexible approach, even multilayered films can be effortlessly prepared. In the glass industry and solar cell production, spray pyrolysis has been used for many decades [98]. An atomizer, precursor solution, substrate heater, and temperature controller are all typical components of spray pyrolysis equipment. The schematic illustration of the spray pyrolysis process is shown in Figure 3a [99]. For the readers interested in a detailed study of this method, please consult [98]. The magnified image of the spray nozzle spraying methanol containing a small amount of HCL is shown in Figure 3b [110]. In [99], a homemade spray pyrolysis technique is used to prepare SnO_2_ thin films by utilizing aqueous solutions of SnCl_4_·5H_2_O. The FESEM micrograph of SnO_2_ thin film is shown in Figure 3c. The deposited thin film shows pores on the surface containing nanoparticles.

*B.2. Sol-gel method*

The sol-gel method, which involves a suspension of colloidal particles, was invented at the dawn of chemistry. The groundbreaking work of Ebelmen (a French chemist) in the 1800s is credited with establishing a sol-gel synthesis of silicon tetra isoamyl oxide from silicon tetrachloride and isoamyl alcohol [111]. The latter study included the synthesis of boron amyloxide, boron ethoxide, and boron methoxide using isoamyl alcohol, ethanol, and methanol, respectively, with boron trichloride [111]. The sol-gel method is a wet chemical method for creating thin-film coatings. Its key benefits are the overall low cost of the procedure relative to more conventional processes such as CVD and PVD, as well as the ability to adapt the composition and properties of thin films to adjust the requirements of the anticipated application [112].

**Figure 4:**
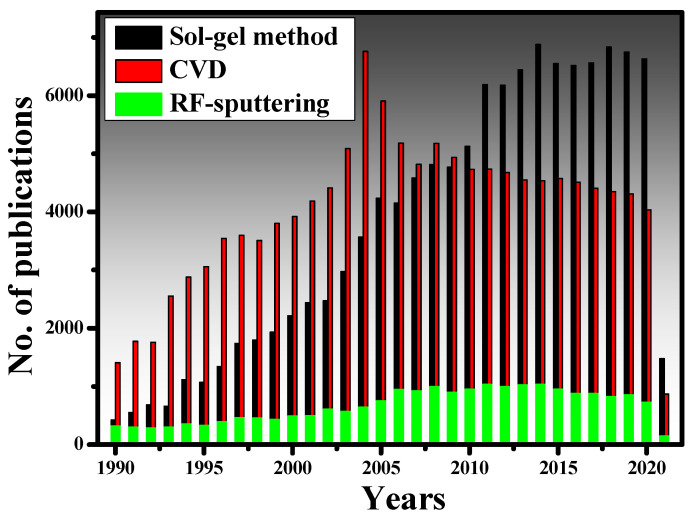
The number of publications related to CVD, RF-sputtering, and sol-gel method, indexed in the Scopus database. The keywords “sol-gel dip coating method”, “Chemical vapor deposition” and “RF-sputtering” were used during the search.

Thin-film coating methods must fulfill the requirements of complete control of film thickness to be successfully used in integrated optics. As a result, thickness management is critical for thin-film development processes in general, and sol-gel is no exception. It has been indicated that the final thickness is primarily determined by coating speed, angle of inclination, and sol concentration. Besides, sol viscosity, density, and liquid-vapor surface tension can also influence the final heat-treated thickness [113]. According to [114], the coating process must be carried out in a cleanroom environment to acquire sol-gel thin films of high optical quality. In [115], a three-step sol-gel process was established to make organic dye-doped thin films with customized porosity for applications in chemical sensing and optoelectronics. Moreover, sol-gel-derived ceramic films are also presented in [116]. More significant works on the sol-gel method can be found here [117,118,119,120,121,122,123,124,125,126]. To understand the popularity of the sol-gel and other traditional methods, we have plotted the number of research papers published on the sol-gel method, CVD, and RF-sputtering from the year 1990 to 2021, as shown in Figure 4. The data has been taken from the Scopus database, which is one of the authentic databases like Web of Science and Google Scholar. From 1990 to 2004, quite intensive research was conducted on CVD while the sol-gel method was still growing. After 2004, the sol-gel method gained more recognition for the deposition of high-quality thin films. The RF-sputtering method is also widely used in research but does not seem to be as prevalent as the other two methods.

### 2.2. Refractive Index Modification Methods

The refractive index (RI) of a material is a number that describes how the light will propagate through it. Light travels at different speeds in the materials having different RI, which can be changed by modifying the density of the material. Here, we have discussed three major methods which can be used to locally modify the RI of the material for the implementation of optical WGs.

#### 2.2.1. Ion Exchange Process

Ion exchange is a primeval method focused on replacing an ion existing in the glass (typically Na^+^) with another ion (e.g., Ag^+^, K^+^, and Li^+^) typically provided by a salt melt [127]. It dates to the first era as a technique for painting glass: it seems that Egyptians previously utilized it in the sixth century to embellish plates and vessels with a brownish-yellow color. The Moors used this method to dye the window glass of their palaces in Spain a few centuries later. In the 1960s, the solidification of the glass surface by ion-exchange transformed into a routine industrial procedure. Since the early 1970s, the appropriateness of ion exchange technology for the development of optical WGs in glass has been known as the groundbreaking works of Izawa and Nakagome [128] and Giallorenzi et al. [129] demonstrated techniques to benefit from the upsurge in the RI of integrated optics created by replacing the Na^+^ with another ion having higher electronic polarizability, for instance, silver (Ag).

Glass is a well-known optical medium, and glass WGs offer several benefits, notably inexpensive material costs, compliance with optical fibers, low propagation loss and birefringence, and good stability and durability. Ion exchange as a fabrication method offers convenience and cost savings because it does not require complex production equipment. It supports batch processing making it adaptable to a wide range of applications. The ion exchange method has also been shown to be suitable for the industrial manufacture of WG components; however, unique fabrication criteria for modules that will be used in the field must be met. After more than 10 years into ion-exchanged WGs, interest has steadily evolved, and basic demonstrations of the practicability of single-mode devices and the process’ adaptability have laid the groundwork for the technology’s prospects, such as a serious commercial production possibility frontier. Findakly examined the state of ion-exchanged glass WG available technology [130].

**Figure 5:**
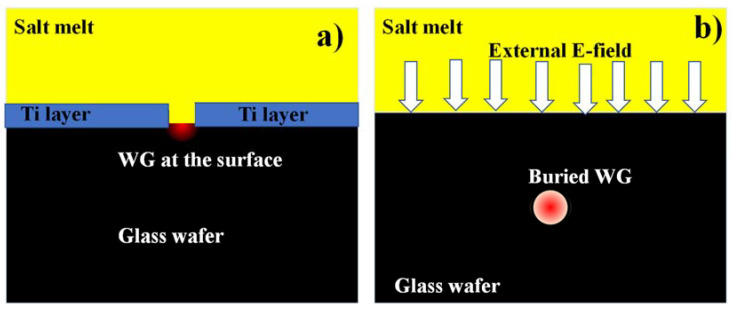
(**a**) Thermal ion-exchange produces a D-shaped surface WG, (**b**) Electric-field assisted buried WG.

Diverse ion exchanges have been studied, such as Ag, K, Cs, Rb, and Tl ions. These processes have been widely employed to manufacture graded-index optical planar and channel WGs [131,132]. They exploited equally ion diffusion from molten salt baths and the electro-migration of a metal film deposited onto the substrate. For some excellent review papers on diverse processes and ion exchange models, please consult [133,134]. Several research groups fabricated ion-exchange WGs for different purposes such as all-optical-switching and sensing [135,136,137,138]. To attain a symmetrical mode field and thus an optimum fit to the modal field of input/output fibers, the burial channel WGs are of particular interest. Simple thermal annealing after ion exchange can be beneficial, but the best outcomes are gained through field-assisted processes [139]. For thermal diffusion and exchange operations, laboratory equipment can be as basic as an oven with an enclosed chamber for the salt melt (Figure 5a). The ability to precisely regulate the operating temperature is critical for process repeatability. Machinery is slightly more complex for field-assisted processes using molten salts, since electrical contacts must be formed to the anode and cathode sides of the substrate, and these must be kept in electrical isolation (Figure 5b). Many examples of process configurations can be found in the WG manufacturing literature; a more extensive explanation of equipment for field-assisted ion exchange can be found in [140].

#### 2.2.2. Ion Implantation

Ion implantation has shown to be an effective method for manufacturing optical WGs in different substrates [141,142]. In the instance of light ions being inserted, the physical density of the substrate is decreased by the damage instigated by nuclear collisions during implantation. As a result, the region with a RI lower than the substrate can function as an optical barrier that allows the light confinement in a thin layer between the surface of the substrate and the optical barrier. However, this optical confinement can be somewhat weak, and the alleged tunneling effect may occur, resulting in the energy leakage of the propagating light. To diminish light leakage to the substrate through the optical barrier, multiple-energy implants are frequently employed to extend the barrier width [143]. Heavy ions, on the other hand, may upsurge the physical density and polarizability of the substrate, which raises the RI of the implanted region bounded by the region with a lower RI, resulting in a typical optical WG. The exact precision of implantation depth and the number of doped ions employed are two main benefits of employing ion implantation. The implantation depth is proportional to the acceleration voltage, which is generally 10–100 keV, while the ion current may be used to measure the number of ions (called the dosage). The ion implantation process is complicated, and the readers who seek in-depth knowledge on this topic are referred to [144].

**Figure 6:**
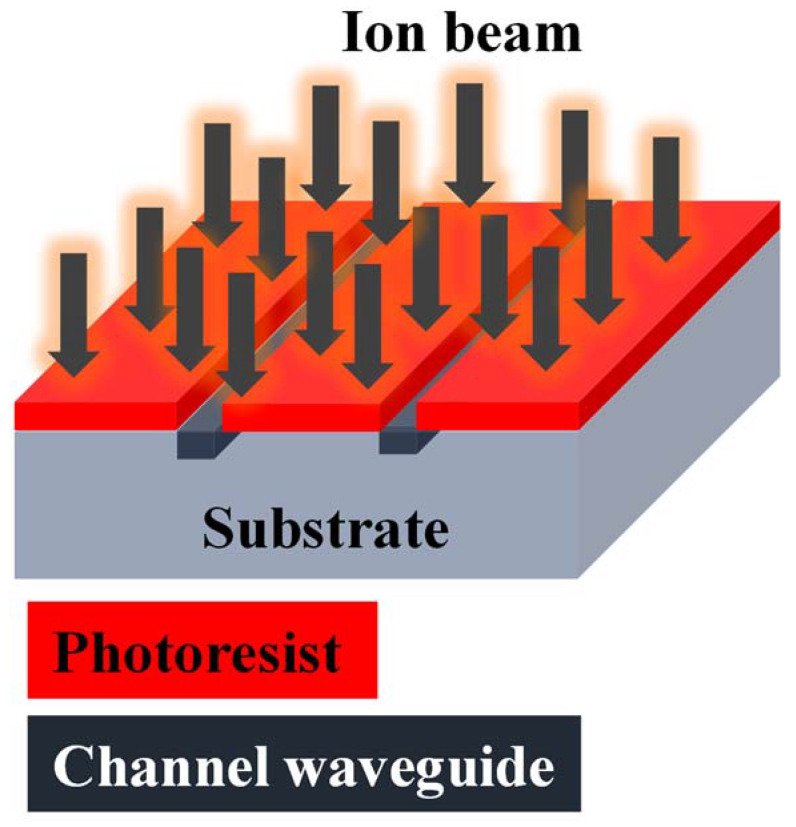
Schematic illustration of the channel WG manufacturing procedure via ion-implantation.

In [145], a new type of optical WG is fabricated by Ge ion implant in the silicon layer on the SOI platform, which can be possibly removed by laser annealing. In [142], the planar and ridge optical WGs have formed in rutile TiO_2_ crystal by He^+^ ion implant merged with micro-fabrication technologies. Planar optical WGs are manufactured by high-energy (2.8 MeV) He^+^-ion implantation with a dosage of 3 × 10^16^ ions/cm^2^ and triple low-energy (450, 500, 550) keV He^+^—ion implantation at room temperature with all fluences of 2 × 10^16^ ions/cm^2^. Jiao et al. demonstrated the optical channel WG in *x*-cut KTiOAsO_4_ crystal produced by photo masking and following direct O^+^ ion implantation at an energy of 3.0 MeV and fluence of 5 × 10^13^ ions/cm^2^. The propagation losses of the WG are 1.2 dB/cm, which displays satisfactory guiding characteristics [146]. Schematic illustration of the channel WG manufacturing procedure via ion-implantation is shown in Figure 6.

#### 2.2.3. Femtosecond-Laser Writing

Integrated optics can be more readily miniaturized and incorporated with micro-electronics resulting in accurate and exceedingly integrated systems, contrary to many optical fiber technologies. In 1996, femtosecond (fs) laser writing was first established and had been thoroughly investigated ever since [147]. Due to its ability to swiftly and flexibly direct the inscription of the complex structures with satisfactory accuracy, fs-laser WG writing in the glass is an encouraging method for integrated optics [148]. Photolithography and FIB micromachining, on the other hand, are slower. In addition, materials, including glasses [149,150], crystals [19,151], and polymers [152,153], WG structures can now be written on directly. Compared to the most popular techniques for the development of WG, fs-laser writing has the advantages of quick production, versatility in WG design, high three-dimensional accuracy, and easy integration of the resulting WG structures with optical fiber E-beam lithography and PECVD.

It is interesting to develop integrated photonics WG sensors that do not involve any etching or complex fabrication procedures. For that reason, the WG should be written near the surface of the substrate. In most previous studies [154,155,156], the WGs were embedded inside the glass substrate via fs-laser writing. However, there are still a few reported studies on the laser-written WGs near the surface of the substrate [157,158,159]. This situation is mostly due to the ablation of glass that happens near the surface while focusing. Writing near-surface WGs is inspired by the desire to allow light to interact with the ambient medium to create integrated sensors in glass chips. Fast fabrication, flexibility in WG design, high spatial precision (i.e., limited by beam quality, wavelength, and polarization), and simple integration of the manufactured WGs with fiberized modules are all advantages of fs-laser direct writing over the most common methods, EBL and PECVD.

**Figure 7:**
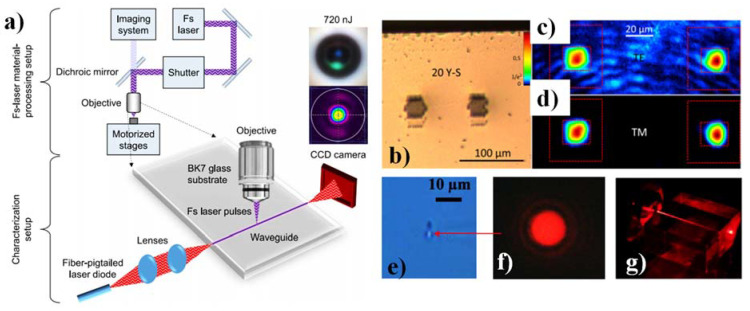
(**a**) Schematic of the fs-laser material processing system and the characterization method [149], (**b**) Microscope image of the Y-splitter laser written in KTP crystal [19], (**c**) Near field pattern of the Y-splitter for TE-polarized light [19], (**d**) Near field pattern of the Y-splitter for TM-polarized light [19], (**e**) Microscope view of the fs-written borosilicate glass WG [160], (**f**) Near field profile of 650 nm red laser light of the same WG [160], (**g**) Camera view of the red laser beam coupling of fs written WG [160].

The fs-laser writing scheme displayed in Figure 7a employed in [149] was constructed with 1047 nm wavelength, 250 fs pulse duration, utilizing a 1.25 numerical aperture infinity-corrected microscope lens with 100 × amplification and a working distance of 460 μm. In [19], a direct low-repetition rate fs-pulse laser was employed for the realization of optical WGs in KTP crystals. In addition, a successful demonstration of a Y-splitter design was reported. The recorded micrograph of the Y-splitter design is shown in Figure 7b. Moreover, the E-field confinement of TE and TM-polarized light is also shown in Figure 7c,d, respectively. TM mode shows better confinement than TE mode [19]. Figure 7e illustrates the optical microscope cross-section picture of borosilicate glass WG written with an average fluence of 39.1 kJ/cm^2^ and a writing speed of 10 mm/s. The arrow suggested that the guiding area is brighter in color [160]. Figure 7f depicts the output facet’s near-field mode pattern while red laser coupling is used with the same WG. The transverse mode was found to be almost circularly symmetric. The mode field width is approximately 2 μm [16]. The camera image of a red laser coupling with a microscope lens of fs written glass WG is shown in Figure 7g [160].

## 3. Author’s Commentary on Silica-Titania Optical Platform Development via a Sol-gel Dip-Coating Method

Due to their potential optical applications, silica, titania, and silica-titania materials obtained by the sol-gel method have been comprehensively studied [35,161,162,163,164,165,166,167,168,169,170,171,172,173]. The origins of their application for fabrication of silica-titania WG films dates back to the first half of the 1980s. Herrmann and Wildman were the first who successfully achieved it. However they did not synthetize sols, but applied commercially available liquicoat solutions provided by MERCK [169]. These WG films deposited on glass substrates using the dip-coating method have refractive n ~ 1.8 (λ = 612.5 μm) and became the material platform for planar evanescent WG chemical/biochemical sensors developed in the research group led by W. Lukosz [170,171,172,174]. The optical losses of those films were ~2.5 dB/cm for λ = 632.8 μm [174]. Jiwei et al. reported fabrication of SiO_2_-TiO_2_ WG films deposited on SiO_2_/Si (111) substrates using the spin-coating method [173]. Those films had maximum refractive index of n ~ 1.87 (λ = 632.8 μm), but their optical losses were quite high ~7.4 dB/cm. It should be mentioned that such a high value of refractive index was achieved for annealing temperatures of 750 °C. One should expect that for such high temperatures the phase transition from anatase to rutile occurs, rendering WG films much more loss-prone. There are also several papers reporting fabrication and characterization of composite SiO_2_-TiO_2_ films, but their waveguiding properties were not examined [162,175,176,177].

**Figure 8:**
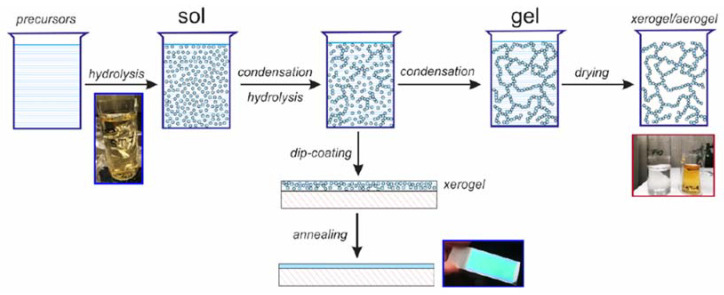
Graphical illustration of the sol-gel fabrication technique for SiO_2_-TiO_2_ WG layers [35].

The sol-gel based fabrication technology of SiO_2_-TiO_2_ is very difficult if the content of titania is larger than 20% wt. That is because titania has the strong tendency to crystallization and formation of separate phases, as a result of which, fabricated films are not amorphous and have high optical losses [178]. Long term stability is another problem which leads to rise of optical losses over time [179]. Our research group managed to overcome these difficulties. We are able to fabricate low-loss, long-time stable SiO_2_-TiO_2_ WG films having TiO_2_ content of 50% wt. [161]. Silica–titania WG layers with a SiO_2_-TiO_2_ = 1:1 molar ratio were formed by applying the dip-coating method on BK7 glass substrates and then heated at the temperature of 500 °C. Tetraethyl orthosilicate Si (OC_2_H_5_)_4_ (TEOS) and tetraethyl orthotitanate Ti (OC_2_H_5_)_4_ (TET) are the main reagents providing the precursors of silica SiO_2_ and titania TiO_2_, respectively. The other reagents employed in the process are water, ethanol, and hydrochloric acid (HCl), catalyzing the reactions of hydrolysis and condensation. A schematic representation of the fabrication technique with photographs of substrates and products of the reaction is shown in Figure 8.

All SiO_2_-TiO_2_ layers are fashioned to be single-mode WGs. In our previous work [180], the technological technique utilized in the formation of the WG layers and the impact of the extraction rate of the substrate on the thickness and RI were shown. The results associated with the uniformity of the chemical composition of the WGs, surface morphology, and optical transmission losses are presented in [161]. The sol-gel method is highly effective and does not involve costly high-tech devices [181,182]. Furthermore, by utilizing the sol-gel process, the RI of the WG layers can be precisely controlled, and the optical losses suffered by these WGs are analogous to the WGs acquired from the LPCVD method [183].

**Figure 9:**
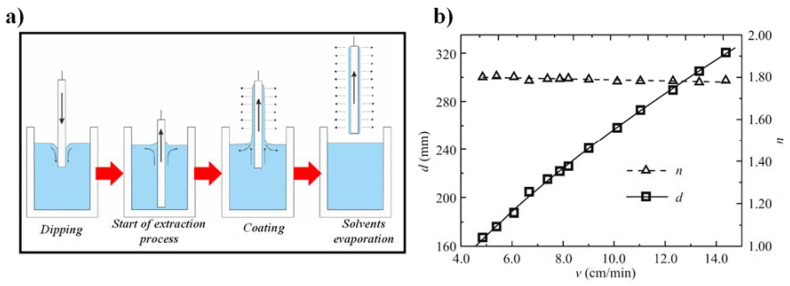
(**a**) Graphical illustration of a dip-coating method for the deposition of SiO_2_-TiO_2_ WG layers [35], (**b**) Dependence of the sample extraction speed from the sol on the WG film thickness and the RI, respectively [180].

Utilizing the dip-coating method, the layers of two-component sol SiO_2_-TiO_2_ were deposited on glass substrates (*n* = 1.509), enabling the precise control of film thickness with the fine-tuning of the substrate extraction speed from the sol [116]. The schematic diagram of the dip-coating method is revealed in Figure 9a. The impact of the sample extraction speed (*v*) on the thickness (*d*) and the refractive index (*n*) of films is shown in Figure 9b. Afterward, the sample was annealed at 500 °C for 60 min. The experimental points are marked with open squares and open triangles [180].

**Figure 10:**
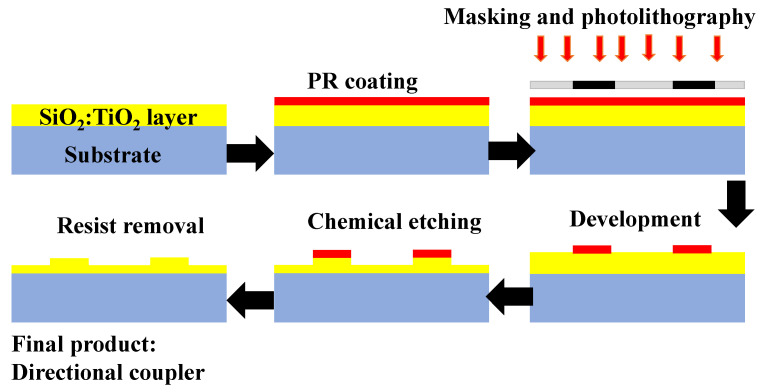
Fabrication steps of DC as mentioned in [184].

In our previous works [183,184,185], rib WGs and directional couplers (DCs) were manufactured using conventional optical photolithography and wet chemical etching in SiO_2_-TiO_2_ WG layers. With the process shown in Figure 10, the DCs were manufactured. The WG layers designed in the sol-gel method were spin-coated with a positive Shipley S1813SP15 photoresist (PR). Soft baking is done before the PR is exposed to UV light through the positive Cr-mask. The development process provides the selective removal of the PR from the SiO_2_–TiO_2_ layer, which facilitates the formation of rib structure during chemical etching. DCs were formed utilizing selective etching of sol-gel derived silica-titania WG films in the solution of ammonia fluoride. The projected WG rib height can be controlled by observing the etching time.

**Figure 11:**
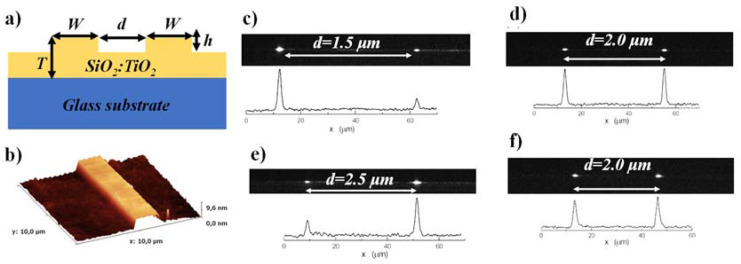
(**a**) Cross-sectional view of the directional coupler based on SiO_2_-TiO_2_ platform, (**b**) Atomic force microscopy of the rib WG. Adapted with permission from [184]. Near-field image of a 1 × 2 DC and related distributed optical power when the coupling length (*L*), (**c**) 1.5 mm, (**d**) 3.0 mm, (**e**) 3.0 mm, (**f**) 4.5 mm. Adapted with permission from [184].

In Figure 11a, a graphical illustration of the DC structure is indicated. Shallow-etched single-mode WG structures (*T* ≈ 180 nm, *h* ≈ 5 nm, *W* = 2 μm) were obtained, having a single-mode propagation range defined by the single-mode propagation condition [184]. The atomic force microscopy (AFM) of the rib WG is shown in Figure 11b. The near field images of the outputs of DCs, as well as optical power distributions corresponding to them, are also shown. Four different cases were explored where the light distribution in DC can be controlled by the WG separation (*d*) and interaction length (*L*). Consequently, a different proportion of the output power, such as 1:0.27, 1:1, 0.42:1, and 0.93:1, is presented at the outputs of distinct DC as shown in Figure 11c–f [184].

## 4. Future Goals and Challenges

The research on the silica-titania platform demonstrates extraordinary attributes such as low-cost WG technology development, low transmission loss, and chemical resistance to oxidation [186]. This opens up the doors for this technology to compete with the existing mainstream integrated photonics platforms, especially in low-volume niche applications such as sensing [187]. The silica-titania PIC technology was designed from the start to be a low-cost option for possible application by SMEs or less-developed research organizations. In comparison to mainstream technology, the proposed innovation can save money on three fundamental steps: (a) WG film procurement (using gaseous phase film deposition, such as LPCVD in the case of SiN and InP, or ordering SOI wafers), (b) lithography (electron-beam or deep UV lithography), and (c) plasma etching (RIE or ICP). Expensive equipment is required for each of these stages. The purpose of silica-titania technology is to replace these procedures with a two-step technique that includes the acquisition of a sol-gel WG film and direct nanoimprinting of the WG pattern. Both procedures are significantly less expensive than the previously indicated three-step procedure, making the suggested SiO_2_-TiO_2_ technology an economically appealing option for SMEs. In Table 1, the features of the three major WG platforms are compared to the SiO_2_-TiO_2_ WG layer technology, which shows the neck-and-neck rivalry among them. When this technology matures, we believe that the SiO_2_-TiO_2_ platform can conquer the existing expensive and difficult-to-handle WG technologies in the longer run.

## 5. Conclusions

Herein, we have reviewed the most desirable and widely used thin-film fabrication techniques along with the advantages/disadvantages associated with each of them. In a few decades, thin-film technology has evolved, offering several methods based on the physical, chemical, and local modification of the RI of the material for the implementation of integrated photonic devices. There is always a need to find cost-effective and easy-to-implement solutions to deposit high-quality thin films over the substrate. As a result, the technology that combines the sol-gel method and dip-coating technique has emerged as a viable solution for the deposition of a high-quality and low-loss thin film in a less complicated way.

Many production methods favor CVD because it is not limited to line-of-sight deposition, which is a common property of sputtering, evaporation, and other PVD methods. As a result, CVD has a lot of throwing power. PVD offers various benefits, including the ability to generate coatings with better qualities than the substrate material, the ability to use all sorts of inorganic and organic materials, and the ability to employ all types of inorganic and organic materials. When compared to other procedures like electroplating, the technique is more ecologically friendly. However, its major limitations are as follows: The PVD method necessitates the use of specialized equipment at a considerable cost. PVD coatings have a slower manufacturing rate than other coating deposition methods. In substrates with complicated geometries, the PVD method is restricted.

Fs-laser inscription has long been established as a significant instrument for engineering a wide range of materials for a variety of purposes. WGs with a variety of designs have been created using fs-laser writing to effectively modify the refractive indices of dielectric crystals. The waveguiding qualities are determined not only by the laser writing settings, but also by the composition of the crystal. Because of the unique properties of fs-laser pulses, technological advances in the field of fs-laser processing have been developed, specifically, in the manufacture of two- and three-dimensional permanent structures within transparent optical materials for use as WGs, photonic crystals, diffraction gratings, beam splitters, and other essential elements in the disciplines of optics, photonics, and communications.

Spray pyrolysis has several advantages, including an open atmosphere process, the ability to monitor the deposition protocol, inexpensive and continuous operation, the absence of high-quality regents as precursors, a high rate of production, manageable crystal size with large surface area, and compositional uniformity of the products.

Ion-exchanged WG technology, according to the authors, will seek to function as an essential platform for testing new ideas and showing novel device designs due to its simple design. Ion-exchanged splitting devices for fiber optic communications may play a key role in commercial applications as markets grow. The technology is also expected to remain a popular choice for several “niche” applications, including WG lasers and a wide range of sensors.

The sol-gel approach has several advantages, including ease of fabrication, good film homogeneity, the ability to cover surfaces of any size and over huge regions, and a low processing temperature. Silica-titania is an interesting and evolving platform for the implementation of active and passive integrated photonic elements which can be employed in optical interconnects and sensing applications. This WG system has not been widely explored as a silicon-on-insulator platform. There is still much room for exploring its physical, chemical, and optical properties to make it an easily accessible platform that can be employed in several interesting applications. This review also presents our recent accomplishments in the silica-titania platform developed via a sol-gel dip-coating method. We believe that our paper will be valuable for researchers working in thin-films technology, especially silica-titania sol-gel dip-coating developed optical elements.

## Figures and Tables

**Table 1:** Comparison of developed SiO_2_-TiO_2_ integrated photonics technology with InP, SOI and SiN technologies [35].

WG Layer	InP	SOI	SiN	SiO_2_-TiO_2_
RI	3.4	3.42	2.0	1.81–2.2
Spectral range [μm]	NIR	1.1–6.5	VIS-NIR	VIS-NIR
Propagation loss (dB/cm)	>0.4	<0.1	<0.1	~0.1
Fabrication method of WG films	LP MOCVD	Wafer bonding	LPCVD	Sol-gel
Implementation costs	High	High	High	Low
Technological maturity	High	High	High	Increasing (under development)
Cost-efficiency	Moderate	Very high	Moderate	Very high
Available integration scale	Very high	Very high	Moderate	Moderate
Tailoring of the RI	No	No	Yes (only for SiO_x_N_x_)	Yes (1.2–2.2)
Applications	Telecommunication	Telecommunication, MEMS, sensors	Telecommunication, MEMS, sensors	Sensors, special applications
Chemical resistance	Low (tendency to oxidation)	Low (tendency to oxidation)	Moderate (tendency to oxidation)	Very high

## Data Availability

Not applicable.

## Abbreviations

Waveguide = WG; Flame hydrolysis deposition = FHD; Chemical vapor deposition = CVD; Silica-titania = SiO_2_-TiO_2_; Physical vapor deposition = PVD; Electron beam = E-beam; Vapor-liquid-solid = VLS; Cathodic arc deposition = CAD; Argon = Ar; Ion beam deposition = IBD; Focus ion beam = FIB; Ion beam assisted deposition = IBAD; Metal-organic chemical vapor deposition = MOCVD; Plasma enhanced CVD = PECVD; Liquid phase deposition = LPD; Refractive index = RI; Atomic force microscopy = AFM; Silicon-on-insulator = SOI; Silicon nitride = SiN; Indium phosphide = InP.
